# The Valuation of Recreational Use of Wetlands and the Impact of the Economic Crisis

**DOI:** 10.3390/ijerph17093228

**Published:** 2020-05-06

**Authors:** Fernando Vidal Gimenez, Claudio Ruiz Mas

**Affiliations:** Agri-Environmental Economics Department, EPSO, Carretera de Beniel, km 3.2, University Miguel Hernández, 03312 Orihuela, Spain; claudioruizmas@gmail.com

**Keywords:** recreational value, wetlands, travel cost method, contingent valuation, willingness to pay, consumer surplus, entrance fee

## Abstract

The economic valuation of environmental resources is of great interest to society in general and to public managers in particular. It can promote more sustainable environmental policies, as it clearly shows the high economic value of natural resources. Thus, these valuation tools can provide useful evidence to support such policies by quantifying the economic value associated with the protection of such resources. However, there is an inherent difficulty in the implementation of methods to assess the economic valuation of environmental resources, mainly as a result of the absence of a market and hence a price that explains its social demand. However, both the travel cost method and the contingent valuation method used in this paper offer an approach to the economic values of the recreational services for wetlands. The aim is to analyze whether these values have been influenced by the economic crisis, so two time periods are compared separated for a decade. Results do not show an unequivocal influence between values in both periods, with different behaviors among natural areas, although with a certain tendency to increase in the decade being analyzed.

## 1. Introduction

Natural resources and the environment in general, have various functions that are highly valued by society [1]. On the one hand, they are part of the production function of a large amount of economic goods, in addition to providing natural goods whose services are demanded by society. On the other hand, they act as a waste receptor service. Finally, the environmental system provides life support services for people [2].

For the last few years, attention has been paid to a new way of organizing economic activity that tries to alleviate the negative effects of traditional economic development models, which threatened both economic stability and the integrity of natural ecosystems. Thus, the model of the Circular Economy emerges, as an alternative to these traditional models, promotes a more rational use of resources to achieve sustainable development where economic, social, environmental or technological aspects are integrated and interacted simultaneously. In short, a greener economy, committed to conservation status of ecosystems and increasing of the ecosystem services potential [3,4,5,6,7,8,9]. Knowledge of ecosystem services and its application in the economy offers a number of benefits (environmental and economic), and as [9] indicates: “Valuating natural capital and the flows of ecosystem services provides a strong economic driver to preserve nature, and to use nature-based solutions to address today’s economical challenges”.

The economic valuation of natural resources means being able to have an indicator of their importance in the welfare of society that allows comparison with other alternative possibilities [10]. This common denominator is none other than money, and monetary valuation does not mean establishing a market valuation. To link a monetary amount with the economic value of an environmental good is not intended to represent a price, but a simple monetary indicator of the value it has for an individual or set of individuals. In other words, what is intended is to reflect in some way what is sacrificed or what is waived to maintain that environmental good. Moreover, since, generally, what is waived is welfare; a common way to measure it is in terms of income. Thus, this economic value does not mean that that amount will subrogate a market price but represents a simple monetary indicator useful for comparative purposes. Therefore, the economic value of environmental goods only has real meaning when defined as a change from another situation: that is, it depends on the context, the situation in the absence of change, and therefore can never be considered as an absolute value [11].

The components that confer value on a natural resource, for example, a wetland, are, on the one hand, a component of use value. It corresponds to the measure of welfare that reports to the individual (society) the active consumption of the resource in one form or another. On the other hand, the non-use value component, which includes all those sources of value that do not imply a current or future use of the environmental good [12].

Traditionally, natural areas have been undervalued given their lower direct profitability purely in economic terms. However, this situation has changed and, in addition to the above, many recreational and landscape services have been recognized, as society has become increasingly aware of the need to protect the environment.

The environmental goods studied here have the particularity of being wetlands. Wetlands are amongst the Earth’s most productive ecosystems. They have been described both as “the kidneys of the landscape” because of the functions they perform in the hydrological and chemical cycles and as “biological supermarkets”, because of the extensive food webs and rich biodiversity they support [13]. Wetland ecosystems provide a diversity of services vital for human well-being and poverty alleviation [14]: “That provisioning services from wetlands, such as food (notably fish) and fiber are essential for human well-being. Supporting and regulating services (such as nutrient cycling) are critical to sustaining vital ecosystem functions that deliver many benefits to people. The delivery of fresh water is a particularly important service both directly and indirectly. In addition, wetlands have significant aesthetic, educational, cultural, and spiritual values and provide invaluable opportunities for recreation and tourism”.

A formal definition of wetland could be as follows [15]: “areas of marsh, fen, peatland or water, whether natural or artificial, permanent or temporary, with water that is static or flowing, fresh, brackish or salt, including areas of marine water, the depth of which at low tide does not exceed six meters”. Wetlands cannot therefore be classified unequivocally as aquatic or terrestrial environments, as they have specific features of both, although they are characterized by a continuous presence of water such that there is an alteration of their soils and therefore of the life they support (flora, fauna, etc.).

**Figure 1:**
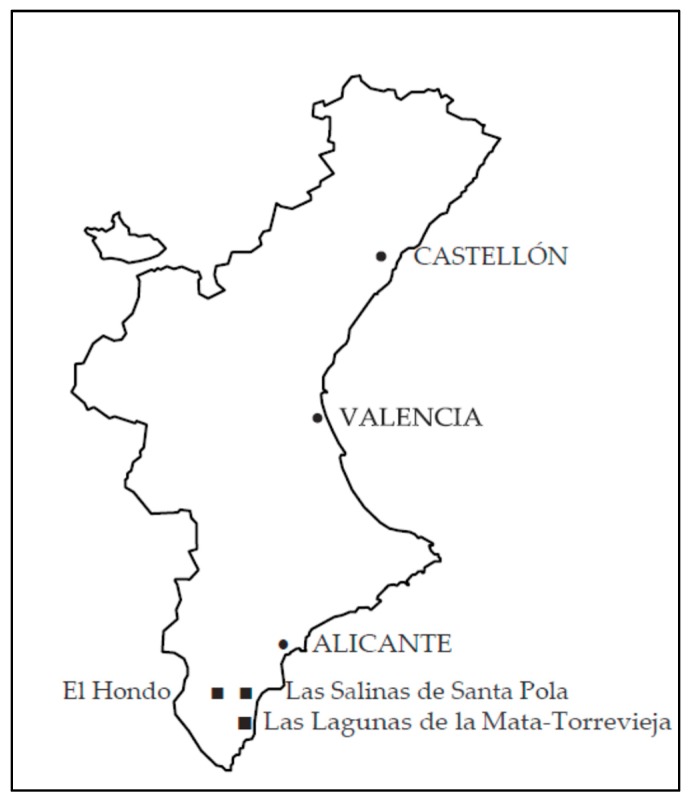
Natural parks located in the southeast part of Alicante province (Valencia Region, Spain).

The province of Alicante (belonging to the Valencian Region in Spain) has six Natural Parks (NPs), three of which will be studied, whose particularity is that of being both wetlands and located in the southeast part of the province (Figure 1). These are El Hondo Natural Park (HNP), La Mata and Torrevieja Lagoons Natural Park (MTNP) and the salt marshes of Santa Pola Natural Park (SPNP). Although there are different methods of environmental valuation, such as the hedonic price and the hedonic wages, the production functions, the market price, the replacement cost, the avoidance cost, choice experiments, etc. for the valuation of the recreational use of these wetlands, the main objective of this work, the travel cost method and the contingent valuation methods will be applied [16,17,18]. These three wetlands have a very marked anthropic character. Direct human actions on them have given them the image they currently have, wetlands at the direct service of human beings, either for successive engineering works to obtain, for example, salt, or water storage for further distribution. They are the only elements that survive from Elche’s original Albufera, reduced in its size by drainage works carried out in the 18th century. Last and not least, they form a triangle of wetlands of exceptional importance for their unique flora and fauna.

At this point, it should be noted that the uniqueness of this paper lies in the length of the study period, covering a decade. The aim is to analyze the effect that the economic crisis, whose beginnings go back more than ten years, has had on the economic valuation given by visitors to these areas [19,20,21,22]. To this end, the results obtained from the questionnaire carried out in 2004/2005, years in which the Spanish economic situation could be rated as optimal, will be compared with a survey in 2013/2014, where the adjective would certainly be quite far from optimal. We are aware that there may be other factors than crisis economic that explain changes in these value (as changes in visitors’ income, labour market situation, alternative recreational areas, etc.), which could be noted as a possible limitation of the study.

The paper is structured as follows: An introduction and objectives of the work are presented in Section 1. In Section 2, all the aspects related with the survey process, the development of the questionnaire and current valuation methods are described. Section 3 discusses the results obtained when applying the travel cost and the contingent valuation methodology, and our main conclusions are given in Section 4.

## 2. Materials and Methods 

The three natural areas analyzed HNP, SPNP and MTNP were declared Natural Parks between 1994 and 1995. All of them appear in the RAMSAR Convention list, also being classified as a Special Protection Area for Birds and Community Interest Sites of the European Commission Natura 2000 Network.

All of them have infrastructure and services for public and touristic use. Table 1 shows both their surface area and the number of visitors during the analyzed decade. As for the temporary distribution of visits, the high seasons are spring and autumn-winter. This pattern is simple to explain as these are the most benign times of the year, with regards to climate and they also correspond to the periods of nesting and hibernation.

As is well known, the objective of economic valuation is to obtain a measurement of the utility and suitability level of the natural environment to meet the needs or to provide welfare to society or its individuals. This value, usually defined as total economic value, is divided into use and non-use values. Use values relate to actual use of the resource, planned use or possible use. The first two cases are very intuitive and easily understandable. The last one, the possible use, it is really an option value, and would reflect how people value to preserve the option of a future use of the environmental good or service. As for non-use values, also known as passive use, they show the willingness to pay for the existence of a good independent of its current, future or potential use. There are different types of non-use values, mainly the following: existence value, altruistic value and bequest value [23,24]. 

The methods that economic analysis provides for environmental valuation try to determine how important the roles performed by the environment are for people. For environmental goods, as mentioned, we are faced with goods that lack a market and, therefore, we find ourselves having to strive either to find a real market with which to relate it or simulate it. 

Thus, and addressing the need set out in the previous paragraph, we could talk about two groups of valuation methods [26]. On the one hand, indirect methods, or revealed preference methods, combining environmental goods, which are the subject of valuation, and goods purchased on the market. When it is impossible to establish links between normal goods (with market) and environmental goods (non-market goods) there is no choice but to resort to direct methods, which are based on what people say about it (declared preferences). Among the indirect methods would be the Travel Cost Method (TCM) while the Contingent Valuation would be among the direct methods (CVM). These two methods will be those used in the economic valuation of the three NP set out above and will both be applied in the two periods under study. In the Environmental Valuation Reference Inventory (EVRI) database [27] there is a huge number of applications of both methodologies worldwide, and their analyses would escape the object of this work. In the Spanish case, the following studies are recommended [28,29,30,31].

The TCM applies not only to the valuation of natural areas but to all resources providing ecosystem services [32,33,34]. These resources play a recreational function in the family utility production function: people visit them for their recreation. Its application is simple. Although in general the enjoyment of NPs is free, visitors incur expenses to be able to enjoy them, a travel cost. It is a question of estimating how the demand for the environmental good varies (i.e., number of visits), from changes in the cost of visiting them [35]. 

The application of this methodology would begin by determining the extent to which a natural area is in demand. To do this, its influence area is defined and the average propensity to visit that space obtained. This will be done in the different zones where area is divided (in the case of its zonal variant, as in the current application). This propensity is compared to the travel cost for each zone, getting an aggregate demand curve for recreational area services. This implicit demand curve will allow us to value, in monetary terms, any change in the offered quantity or quality of these services, by analyzing the changes to the net consumer surplus (visitors). The consumer’s net surplus (CS) is used here as a measure of the welfare earned by a person when he or she acquires a good at a certain price [29,32,33,34]. 

Considering the costs, there are some that are unavoidable, and they are the ones that are directly related to travel. However, there are others which are not as clearly defined as travel costs (i.e., eating or overnight stays), which will not be taken into account if they are pursued and in any case, the differential as opposed to the absolute costs would be taken into account. Another element to consider is the inclusion of the time element, whether it is travel, stay, etc. [36,37,38]. 

Regarding the CVM, it is based on the information provided by the people when they were asked about the valuation under analysis, a particular case within market-building procedures. CVM attempts to measure changes in people’s welfare due to an increase or decrease in the quantity or quality of a good. This measure, in monetary units, is usually expressed in terms of the maximum amount a person would pay for a good, what is often known by willingness to pay (WTP). Alternatively, CVM allows you to find the maximum willingness to accept compensation for a good loss. One of the main differences, and distinct from other methods, is the fact that in addition to the values that users perceive when consuming the good, the user can also obtain welfare or satisfaction even if he/she is not a direct user or consumer of the good [39]. These non-use values, also called passive use, cannot be detected using indirect methods, such as the TCM [40].

The most commonly used vehicle to simulate a market for an environmental good would be conducting a survey. The surveys (questionnaire) should be properly structured, trying to incorporate really important questions for the implementation of the methods, bearing in mind that each survey method used, as well as the format of the chosen questions, has its advantages and disadvantages [40,41]. 

The final questionnaire used in this work consisted of 48 questions, structured in four blocks. The first would correspond to introductory questions, to make contact with the interviewee. The second and third would correspond to questions related to the TCM and CVM, while the fourth included questions related to the most relevant socio-economic characteristics of the visitor. It should be noted that ten visitors from each NP were previously pretested, correcting some detected deficiencies.

A first part of the fieldwork (i.e., surveys of visitors to these natural areas), was done between March 2004 to September 2005 (P_1_). Later, for most of 2013 (February to December) and in 2014 (until the end of July), visitor surveys were carried out again (P_2_). As noted, our objective is to analyze whether the temporary component could influence the valuation, and in particular if, as a result of the economic crisis that we have suffered for over a decade, the economic valuation of these three wetlands has varied.

Surveys were conducted on a randomly chosen sample of visitors within the three wetlands. All respondents were of legal age. A personal interview was chosen, and surveys were conducted in the two periods indicated above. In the number of surveys conducted (Table 2) relevant data relating to the population under study in each NP was taken into account, that is, its number of visitors/year, data obtained from the management reports of each NPs [25]. In the case of P_2_ surveys, to note that in the case of HNP it was not possible to achieve a higher rate of participation, given the complicated relationship between the regional administration and the owner of most of its area (Levante Irrigation Community), which resulted in the closure of routes on certain days of the week, in addition to the problems that the public company responsible for the maintenance and management of the NP was experiencing.

## 3. Results

### 3.1. Travel Cost Method

In the analysis of the demand function, the TCM has been chosen in the non-equidistant zonal variant, differentiating visitors and grouping them by concentric zones around the NP. In the two periods under study, the same number of concentric zones have been selected for each park, while the choice of their amplitude has been made according to the best fit for the initial function. The distance from a central point in the NP to the place of origin of visitors will be considered. These distances will vary among parks and will not necessarily be equidistant (as can be seen in Table 3, Table 4 and Table 5).

The number of visitors for each zone and period is obtained from the survey, while the population census of the National Statistical Institute was used to obtain population data from the municipalities located in each area [42]. Thus, the ratio between visitors and the total population of each area is calculated, such ratio representing the propensity to visit the natural area in each zone.

To calculate the travel cost, the function of each distance traveled to reach the park (kilometers) and the time taken (minutes) has been obtained. Thus, for each distance traveled, the fuel cost per person and kilometer has been calculated, taking into account the weight of gasoline and diesel vehicle pool, the price in euros per liter of each fuel and the average fuel consumption for each period [43]. The average number of occupants per vehicle has also been considered. In cases where visitors have travelled by an alternative means of transport to his own vehicle, the corresponding ticket cost or the usual rate per day for a rental car would be calculated. Meals and accommodation costs are not included as travel costs, due to insufficient and incomplete information collected.

One element of interest is the cost of travel time, that if not considered would be an underestimation of the total effort made for visitors. However, there is no consensus in literature on how to set the value of visitors’ time. Here, and even though most of the sample considers the travel time as enjoyable, the opportunity cost of travel time has been included, in an attempt to increase the different valuation scenarios. Thus, after a review of the studies carried out in Spain, three scenarios have been defined for each period:

- Cost Scenario 1 (S1): Includes only the fuel cost (FC) per person and zone (round-trip cost).
- Cost Scenario 2 (S2): Opportunity time cost (round-trip cost), estimated at 0.06 €/km (OTC1) will be added to the fuel cost (FC)
- Cost Scenario 3 (S3): Similar to S2, except that the opportunity time cost is estimated at 0.14 €/km (OTC2).

To obtain the demand function, in this case the total number of visitors depending on the price of a recreational visit (travel cost), visitors have been considered to react to a variation in the entrance fee in the same way as they would to an increase in the average travel cost [26]. It is not really an entrance fee, but rather the extra travel cost visitors would be willing to spend to access the NP [44]. It requires increasing the travel cost, adding the value of that hypothetical fee and calculating the number of visitors and the propensity to visit from each zone considering the different scenarios [45]. Although not considered here, it is usual to find analysis where the demand function estimate includes along with the number of visits and the travel costs, socio-economic indicators such as income, sex, age, etc. [32,33,34]. 

Table 3, Table 4 and Table 5 show the zones being considered in each wetland, as well as the number of visitors surveyed, the weighted average distances and times used by visitors in both periods and the average propensity to be visited.

Table 6, Table 7 and Table 8 show the cost scenarios that will allow us to determine the points of the initial demand function for each NP and period.

The next step is to calculate the transformed demand function, based on the initial demand function, assuming that visitors react to an increase in the entrance fee in the same way as in an increase of the average travel cost. The procedure for getting the points of the transformed demand function (entrance fee and number of visits) is simple and has been used extensively in literature. It consists of assuming several values for the hypothetical entrance fee, starting from a zero increase in it [28]. Table 9, Table 10 and Table 11 show the transformed demand function points for each NP and Period.

Starting from a zero price in identifying the points of the transformed demand function, the visitor’s surplus would be directly equivalent to the area encompassed by that function. This area represents the total respondents’ surplus, so dividing this amount between them results in the visitor’s surplus (€/person). Table 12 shows this surplus (CS) by scenario and period.

Starting with the HNP, in the most conservative scenario, that is considering only the travel costs (S1), the CS in the first period is just over one euro per visitor, while a decade later this surplus increases by almost twenty per cent, to €1.27/person. By including the opportunity travel time cost (S2 and S3), the CS increases considerably (Table 12); however, this value decreases between periods.

However, the SPNP behavior has been very different. Although as in the previous case, the CS increases among scenarios (by increasing the opportunity cost), it also increases between periods, reaching similar values to those of the previous park. Finally, the MTNP has the lowest CS values and a different behavior to the other two wetlands: it increases between periods for the first two scenarios and decreases in the third. It should be noted that values obtained for the three wetlands, compared to other applications of this methodology in Spain, have a smaller surplus [28,29,31,45,46,47].

Finally, one last exercise could be done to calculate the total recreational use value for each park (Table 13). To do this, we simply multiply the surplus calculated in each scenario by the number of visitors in each period, obtaining a first approximation of the park annual recreational value. This amount would be capitalized indefinitely using a social rate that would include the temporary preferences of the community that values them. This rate, in line with some of the applications in the European environment, would be estimated at 2% per year [28,48]. Thus, the total recreational use value of these NPs would range from 440,000 euros of the HNP in the first period and the 2.58 million MTNP in the second. 

### 3.2. Contingent Valuation Method 

The TCM implementation is complemented using Contingent Valuation (CVM). The interviewee has been asked, in this case as a potential good demander, whether he would be interested in visiting the park, before the interviewer´s proposal (acting as a potential offeror), and if so, how much would he be prepared to pay. Always from the premise that the interviewee intends to increase his well-being. This is a declared preference, that is, how much they would be willing to pay (WTP) as an entrance fee to the different NPs.

As a result of the CVM application there are significant differences in the entrance fee among parks and periods. In addition, it must be pointed out that the percentage of interviewees willing to pay an entrance fee increases significantly between periods for the three NPs (Table 14).

Firstly, we should emphasize that HNP is one of the three studied parks that has higher entrance fees, with average values that can be double those declared in the other two. The average WTP in P_1_ was €4.16, while a decade later it increased by ten percent, to €4.58. This behavior is contrary to the rest of the NPs, where ten years later the WTP is descending significantly. The mode remains constant in both periods (€3), while the median is reduced from €5 to €3 (Table 14). The WTP values in both periods are in line with those registered in Spain for similar studies [28,29,30,45,46,47]. 

The MTNP has much lower WTP values than the PNH, with a reduction of about a quarter, from €2.70 a decade ago to €2. However, neither mode €1, nor median €2, change between periods (Table 14). The behavior in the SPNP is very similar to that seen in the PNMT, with initial WTP values of €2.5 which are reduced by twenty percent a decade later to €1.94, with a median that also drops from €2 to €1, while mode remains at €1 (Table 14). The values obtained for both NPs are low compared to those recorded in the literature in Spain [28,29,30,45,46,47,48,49,50].

It is necessary to keep in mind that when the interviewee responds “nothing” in his WTP, this may be because the interviewee does not really want to declare his true value and may therefore be showing his rejection to how such a question has been proposed. That is, he may not accept the proposed means or payment vehicle. These kinds of responses are known as protest bids and should be distinguished from those answers that show a zero value in terms of their WTP, because if such separation did not take place, the WTP of the aggregate would be distorted. As can be seen (Table 14), and discarded the legitimate zero bids [51,52], two per cent in the worst-case scenario, we see that protest bids are significantly reduced between periods for all wetlands; especially for MTNP and SPNP (although in the latter they are still majority). The main reasons for rejection include the fact that it was a “Public Good” in P_1_, while after a decade the “Lack sufficient income” is more mentioned, followed by “Paying enough taxes”. 

As was done from CS in the TCM, the total recreational value for each park can be obtained, by multiplying the WTP declared by respondents by the number of visitors in each period (data extracted from Table 2) and the capitalized value is a function of a social rate of 2% (and unlimited capitalization as in TCM case). This would provide recreational values of between 1.15 million in the SPNP and 2.65 million for MTNP (Table 15).

In an attempt to model the recreational value of these parks, we try to find a relationship between the willingness to pay an entrance fee for visitors surveyed (WTPEF) and the fee obtained directly from the survey. To this end [28,30,39,52] a binary logistic regression was chosen (Logit):

$$\mathrm{WTPEF}=\frac{1}{1+e^{-\left(a\;+\;b\;\mathrm{FEE}\right)}}+u$$

where WTPEF: Dichotomous variable (1 = willing to pay the fee; 0 = unwilling); FEE: WTP declared by visitors from the prices offered to respondents; a and b: Parameters to estimate (β, Table 16, Table 17 and Table 18) and u: Error term.

In the WTPFE calculation, protest bids have been eliminated [46,53,54,55]. The parameters of these regressions for each NP are shown in Table 16, Table 17 and Table 18. As is well known, in the adjustments of the logistic models, the coefficient of determination (R^2^) cannot be calculated in the same way as in linear ones, so in this case the Cox and Snell R^2^ and the Nagelkerke R^2^ have been used as an approximation. In all three NPs and for both periods these coefficients exceed the recommended 0.15 [54]. In no case have there been any significant differences between the observed and predicted classifications (Hosmer-Lemeshow Test), which would reflect the goodness of fit of the analysis. Moreover, the percentage of correct predictions exceeds 69% in the worst-case scenario.

Thus, in the case of the HNP the mean WTPEF obtained for visitors is €5.47 in P_1_ and €5.62 in P_2_ (Table 16). These values exceed the WTP previously obtained by more than one euro (Table 14). In the MTNP, the logit analysis offers an average entrance fee of €3.98 in P_1_ and €2.43 in P_2_ (Table 17), values that again exceed the WTP obtained earlier, although this difference is reduced by P_2_.

The SPNP shows an average WTPEF (Table 18) of €3.85 (P_1_) and €2.15 (P_2_), values that exceed that obtained earlier (Table 11), although as in the MTNP, this difference is significantly less in P_2_.

## 4. Conclusions

The economic valuation of natural and environmental resources is of great interest to society in general and to public space managers in particular. However, no one escapes the inherent difficulty in the application of economic valuation methodologies to environmental resources motivated by the absence of a market and therefore a price that explains its social demand.

However, both the Travel Cost Method and the Contingent Valuation used here allow us to approach the economic values of the recreational services for the Natural Parks located in the southeast of the Alicante province, three wetlands of great importance in both the Valencian Region and Spain.

As regards the application of the TCM, five concentric zones and three cost scenarios have been raised for each NP. Although an exercise has been done to try and calculate the annual recreational value per PN, it is very dependent on the number of visitors to each park and given the information available, we prefer to focus on highlighting only the Consumer Surplus values per person and period. These values, in this case use values, are usually lower than those of other applications in Spain.

Thus, the CS has been calculated for each scenario and period, so that in HNP and for S_1_ this value is €1.07/person in P_1_ and increases up to €1.27/person a decade later. However, this 20 percent growth between periods cannot be extrapolated to the other two scenarios. The CS value in S2 remains virtually constant (€2.70/person in P_1_ and €2.65/person in P_2_), while in S3 it is reduced by fifteen per cent, from €5.32/person to €4.5/person.

In the PNSS, and for the first scenario, the CS is € 0.78/person, increasing a decade later more than fifty percent, to €1.22/person. In S2 this increase exceeds forty per cent (from €1.78/person to €2.55/person), while in S3 this increase is close to thirty-five per cent €4.33/person in P_2_ versus €3.23/person in P_1_). These values are in all scenarios and periods lower than those of the HNP.

MTNP has the lowest values in terms of CS. These values in S1 range from €0.45/person in P_1_ to €0.60/person in P_2_; in S2 these values increase from €1.14/person to €1.22/person, while in S3 it remains around €2.10/person.

As regards the recreational value using Contingent Valuation, in this case the willingness to pay an entrance fee (WTP), it must be pointed out that the HNP has the highest values (practically doubling the rest), in addition to being the only one where this WTP grows between periods, from 4.16 euros (P_1_) to 4.58 euros (P_2_). It is also worth noting that the percentage of visitors who are likely to pay a fee for visiting the HNP grows (69% in P_1_ and 74% in P_2_). The WTP values are clearly higher than the CS values obtained in S1 and S2 of the TCM application. Moreover, note that these WTP values are in line with other works in Spain.

The WTP values for the MTNP and SPNP follow similar patterns, decreasing by nearly a quarter in the studied decade. In the PNMT it decreases from €2.70 to €2.02, while in the PNSS it decreases from €2.5 to €1.94. If these values are compared with those of TCM, the WTP in PNMT exceeds the S1 surplus and it is clearly lower than that S3, while in the PNSS it exceeds the surpluses obtained for the first and second scenarios and is around S3 surplus. In both parks, despite the WTP reduction, the percentage of visitors who are willing to pay a fee grows. The WTP values in both parks are below those obtained in other similar works in Spain.

The protest bids are reduced in the analyzed decade in three NPs. The main reason for rejection in P_1_ is to be a “Public Good” while in the second period appears “Lack sufficient income” and “Paying enough taxes”, which could be explained by the context of the economic crisis at the time.

Attempts were made to model the recreational use value of these NPs through a logistic regression, which provides a Willingness to Pay for an Entrance Fee by visitors (WTPEF). The obtained values, eliminating protest bids, show a higher WTPEF than the WTP, although its evolution between periods is similar. In the case of the HNP, the values are €5.47 (P_1_) and €5.62 (P_2_); for the PNMT €3.98 (P_1_) and €2.43 (P_2_); while for the PNSS the values are €3.85 (P_1_) and €2.15 (P_2_).

From the above results, we cannot conclude that the economic crisis had a unique and direct influence on the economic valuation of the recreational aspects of these wetlands. In some parks, the crisis appears to have led to lower valuation, but in others the opposite is true. In all cases, visitors are more willing to positively value the recreational aspects of the parks, when this is not linked to economic aspects but rather a greater social awareness towards the environment.

Some limitations and weaknesses of the used valuation methods are, for the CVM: the embedding effect, the ordering problem, the differences with the payment vehicle chosen, the response biases, etc. For the TCM, there are problems related to multi-purpose or multi-activity trips, the definition of the opportunity cost associated with the travel time, the availability of alternative areas, the assumed responses to changes in price, the trip value, etc.

A limitation of our work is that we have considered the existence of the economic crisis as the only element with possible incidence in the WTP. Other factors could explain changes in these values: economic aspects such as changes in visitors’ income or the labour market situation, the offer of alternative recreational areas, changes in parks’ infrastructure, etc. Further analysis should be carried out to include them and to complement what is exposed here.

Another interesting approach could investigate the use of the income as other indicator to be included in the analysis. Thus, in obtaining the demand function, along with the number of visits the different adjustments could include both travel costs and socio-economic indicators as income.

Given the variations that the discount rate may experience and its close connection with the economic conditions of the moment, it might be useful for future use to resort to sensitivity analysis with alternative discount rates to the one used here.

Finally, one of the aspects with potential social relevance and the result of subsequent works would be the comparison of the visitors (“visitor”) values to these NPs, in particular their consumer surplus or their willingness to pay an entrance fee with the costs assumed by the public administrations when it comes to offering their recreational aspects.

This recreational value should also be expanded in the future with the total economic valuation of these spaces (direct use value, indirect use value, option value, existence value, etc.), also including the opportunity costs of non-use for alternative uses in that valuation.

## Figures and Tables

**Table 1:** Area (Ha) and number of visitors to natural parks in southern Alicante.

	HNP	MTNP	SPNP
Area (Hectares)	2387	3700	2491
Visitors/year			
2003	7800	18,073	12,291
2004	8634	21,269	14,778
2005	7493	19,270	13,931
2006	9549	12,217	17,040
2007	8213	12,402	47,310
2008	8774	12,756	46,125
2009	10,094	14,508	62,144
2010	12,516	17,593	41,838
2011	9112	14,278	30,836
2012	9999	15,077	11,909
2013	8774	12,756	46,125

HNP: El Hondo Natural Park. MTNP: La Mata and Torrevieja Lagoons Natural Park. SPNP: Santa Pola Natural Park. Source: [25].

**Table 2:** Number of surveys for natural park (NP) and period.

NP	P_1_ (2004/2005)			P_2_ (2013/2014)		
	Visitors/Year *	No. Surveys	Error Level	Visitors/Year **	No. Surveys	Error Level
HNP	8217	596	3.87%	9999	152	7.89%
SPNP	13,539	541	4.13%	11,909	302	5.57%
MTNP	19,671	501	4.41%	15,077	304	5.56%

Note: Confidence level: 95%; *p* = *q* = 50. * Average visitors for year 2004 and 2005. ** Last available data [25].

**Table 3:** Weighted Average Distance (WAD) and Weighted Average Time (WAT) taken by visitors of HNP for both periods.

Zone	P_1_				P_2_			
	Visitors	WAD (km)	WAT (min)	Visitors/000 Inhabitants	Visitors	WAD (km)	WAT (min)	Visitors/000 Inhabitants
1 (0–10 km)	93	8.92	16.48	1.012355086	10	14.3	18.60	0.09330795
2 (11–20 km)	236	20.15	28.83	0.607871420	77	21.2	29.36	0.17209473
3 (20–30 km)	101	32.72	32.77	0.532914037	22	30.0	33.41	0.09789090
4 (31–50 km)	123	42.43	39.09	0.103805790	32	49.4	44.16	0.02456489
5 (51–80 km)	11	76.82	57.82	0.012051493	1	88.0	60.00	0.00100594
Total	564				142			

**Table 4:** Weighted Average Distance (WAD) and Weighted Average Time (WAT) taken by visitors of SPNP for both periods.

Zone	P_1_				P_2_			
	Visitors	WAD (km)	WAT (min)	Visitors/000 Inhabitants	Visitors	WAD (km)	WAT (min)	Visitors/000 Inhabitants
1 (0–5 km)	86	5.00	4.97	3.373342747	31	3.00	10.00	0.90818539
2 (6–15 km)	45	15.00	19.00	0.190310248	85	16.67	26.91	0.32708788
3 (16–25 km)	93	21.86	32.19	0.162313579	16	20.00	37.81	0.02472520
4 (26–50 km)	46	32.33	44.26	0.088162126	13	32.92	46.38	0.02197100
5 (51–150 km)	3	88.00	66.67	0.002348353	30	76.17	70.90	0.02200586
Total	273				175			

**Table 5:** Weighted Average Distance (WAD) and Weighted Average Time (WAT) taken by visitors of MTNP for both periods.

Zone	P_1_				P_2_			
	Visitors	WAD (km)	WAT (min)	Visitors/000 Inhabitants	Visitors	WAD (km)	WAT (min)	Visitors/000 Inhabitants
1 (0–6 km)	171	6.00	8.00	3.373342747	120	6.00	8.00	1.14063020
2 (7–15 km)	60	11.48	12.62	0.190310248	40	10.95	11.70	0.50467455
3 (16–25 km)	5	21.40	21.20	0.162313579	2	21.00	21.00	0.01077778
4 (26–50 km)	49	34.88	37.67	0.088162126	42	36.95	38.38	0.02673116
5 (51–75 km)	2	68.50	51.50	0.002348353	2	62.00	51.00	0.00256969
Total	287				206			

**Table 6:** Initial demand function points for the HNP (P_1_ and P_2_).

Zone		P_1_			P_2_	
	Scenario 1 FC	Scenario 2 FC + OTC1	Scenario 3 FC + OTC2	Scenario 1 FC	Scenario 2 FC + OTC1	Scenario 3 FC + OTC2
1	0.67	1.74	3.17	1.57	3.29	5.57
2	1.51	3.93	7.15	2.33	4.87	8.26
3	2.45	6.38	11.61	3.29	6.89	11.69
4	3.18	8.27	15.06	5.42	11.35	19.26
5	5.76	14.97	27.26	9.66	20.22	34.30

**Table 7:** Initial demand function points for the SPNP (P_1_ and P_2_).

Zone		P_1_			P_2_	
	Scenario 1 FC	Scenario 2 FC + OTC1	Scenario 3 FC + OTC2	Scenario 1 FC	Scenario 2 FC + OTC1	Scenario 3 FC + OTC2
1	0.37	0.97	1.77	0.33	0.69	1.17
2	1.12	2.92	5.32	1.83	3.83	6.50
3	1.64	4.26	7.76	2.20	4.60	7.80
4	2.42	6.30	11.47	3.61	7.57	12.83
5	6.59	17.15	31.23	8.36	17.50	29.69

**Table 8:** Initial demand function points for the MTNP (P_1_ and P_2_).

Zone		P_1_			P_2_	
	Scenario 1 FC	Scenario 2 FC + OTC1	Scenario 3 FC + OTC2	Scenario 1 FC	Scenario 2 FC + OTC1	Scenario 3 FC + OTC2
1	0.45	1.17	2.13	0.66	1.38	2.34
2	0.86	2.24	4.08	1.20	2.52	4.27
3	1.60	4.17	7.60	2.31	4.83	8.19
4	2.61	6.80	12.38	4.06	8.49	14.40
5	5.13	13.35	24.31	6.81	14.25	24.17

**Table 9:** Transformed demand function points for the HNP (P_1_ and P_2_).

P_1_				P_2_			
Entrance Fee Scenario 1	Entrance Fee Scenario 2	Entrance Fee Scenario 3	Visitors	Entrance Fee Scenario 1	Entrance Fee Scenario 2	Entrance Fee Scenario 3	Visitors
0	0	0	564	0	0	0	142
0.84	2.19	3.98	296.69	0.76	1.58	2.69	69.07
1.78	4.64	8.45	91.54	1.72	3.61	6.12	21.71
2.51	6.53	11.89	14.21	3.85	8.07	13.68	3.08
5.09	13.23	24.10	1.11	8.09	16.94	28.73	0.11
7	15	26	0	9	18	30	0

**Table 10:** Transformed demand function points for the SPNP (P_1_ and P_2_).

P_1_				P_2_			
Entrance Fee Scenario 1	Entrance Fee Scenario 2	Entrance Fee Scenario 3	Visitors	Entrance Fee Scenario 1	Entrance Fee Scenario 2	Entrance Fee Scenario 3	Visitors
0	0	0	273	0	0	0	175
0.75	1.95	3.55	95.0	1.50	3.14	5.33	44.8
1.26	3.29	5.98	26.3	1.87	3.91	6.63	20.8
2.05	5.33	9.70	2.8	3.29	6.88	11.66	6.5
6.22	16.18	29.46	0.1	8.03	16.81	28.52	0.8
7	17	30	0.0	9	17	29	0.0

**Table 11:** Transformed demand function points for the MTNP (P_1_ and P_2_).

P_1_				P_2_			
Entrance Fee Scenario 1	Entrance Fee Scenario 2	Entrance Fee Scenario 3	Visitors	Entrance Fee Scenario 1	Entrance Fee Scenario 2	Entrance Fee Scenario 3	Visitors
0	0	0	287	0	0	0	206
0.41	1.07	1.95	102.22	0.54	1.14	1.93	62.95
1.15	2.86	5.47	5.07	1.65	3.45	5.85	3.73
2.16	6.17	10.25	3.05	3.40	7.11	12.07	3.02
4.68	11.36	22.18	0.24	6.15	12.87	21.83	0.27
5	13	24	0	7	15	25	0

**Table 12:** Natural Parks´ Consumer Surplus (€/person) for Scenario and Period.

Scenarios	HNP		SPNP		MTNP	
	P_1_	P_2_	P_1_	P_2_	P_1_	P_2_
Scenario 1	1.07	1.27	0.78	1.22	0.45	0.60
Scenario 2	2.70	2.65	1.78	2.55	1.14	1.22
Scenario 3	5.32	4.50	3.13	4.33	2.11	2.08

**Table 13:** Natural Parks´ Recreational Value (€) for Scenario and Period.

Parks	Recreational Value	Period	Scenarios		
			Scenario 1	Scenario 2	Scenario 3
HNP	Annual Recreational Value (€/year)	P_1_	8792	22,186	43,714
		P_2_	12,668	26,509	44,964
	Total Recreational Value (€)	P_1_	439,600	1,109,300	2,185,700
		P_2_	633,400	1,325,450	2,248,200
SPNPP	Annual Recreational Value (€/year)	P_1_	10,560	24,099	43,731
		P_2_	14,538	30,368	51,566
	Total Recreational Value (€)	P_1_	528,000	1,204,950	2,186,550
		P_2_	726,900	1,518,400	2,578,300
MTNP	Annual Recreational Value (€/year)	P_1_	8852	22,425	41,506
		P_2_	9046	18,394	31,360
	Total Recreational Value (€)	P_1_	442,600	1,121,250	2,075,300
		P_2_	452,300	919,700	1,568,000

**Table 14:** Willingness to Pay for an entrance fee (WTP) to visit the Natural Parks (€).

Natural Park	Period	WTP (No. Visitors)			WTP (€)					
		Yes	No	NK/NA	Mean	Median	Mode	Std Dev	Min	Max
HNP	P_1_	412	181	3	4.16	5	3	2.69	0	20
	P_2_	112	26	14	4.21	3	3	0	20	3
MTNP	P_1_	166	323	12	1.97	1	2	0	10	2
	P_2_	196	81	27	1.27	1	2	0	10	2
SPNP	P_1_	303	234	4	1.75	1	2	0	10	2
	P_2_	222	47	35	1.21	1	1	0	5	1

**Table 15:** Total Recreation Value (€) for Alicante´s province Natural Parks in both periods.

Natural Park	WTP (€)		Annual Recreational Value (€/year)		Total Recreational Value (€)	
	P_1_	P_2_	P_1_	P_2_	P_1_	P_2_
HNP	4.16	4.58	34,183	45,795	1,709,136	2,289,711
MTNP	2.70	2.02	53,112	30,456	2,655,585	1,522,777
SPNP	2.50	1.94	33,847	23,103	1,692,375	1,155,173

**Table 16:** Logistic regression results for WTPEF in the HNP case.

Variables		β	S.E.	Wald	Exp. (β)	C.I. 95% Exp (β) Inf. Sup.	
P1	FEE (b)	−0.852 *	0.107	62.917	0.427	0.346	0.527
	Constant (a)	4.662 *	0.491	90.156	105.833		
Cox & Snell R^2^: 0,183; Nagelkerke R^2^: 0.276 (−2LL: 352.347) ** Overall correct percentage: 77.4%							
P2	FEE (b)	−0.793 *	0.104	58.501	0.452	0.369	0.554
	Constant (a)	4.458 *	0.480	86.199	86.290		
Cox & Snell’s R^2^: 0,165; Nagelkerke R^2^: 0.247 (−2LL: 366.607) ** Overall correct percentage: 75.5%							

Note: * Significant differences for a maximum error level of 1%; Degrees of freedom (d.f.) = 1; ** There are no significant differences (Hosmer-Lemeshow Test).

**Table 17:** Logistic regression results for WTPEF in the MTNP case.

Variables		β	S.E.	Wald	Exp. (β)	C.I. 95% Exp (β) Inf. Sup.	
P1	FEE (b)	−0.756 *	0.137	30.534	0.469	0.359	0.614
	Constant (a)	3.011 *	0.569	28.020	20.315		
Cox & Snell R^2^: 0.183; Nagelkerke R^2^: 0.276 (−2LL: 186.930) ** Overall correct percentage: 70.4%							
P2	FEE (b)	−1.169 *	0.180	42.030	3.218	2.260	4.581
	Constant (a)	2.829 *	0.406	48.514	0.059		
Cox & Snell R^2^: 0.165; Nagelkerke R^2^: 0.382 (−2LL: 207.641) ** Overall correct percentage: 72.9%							

Note: * Significant differences for a maximum error level of 1%; d.f. = 1; ** There are no significant differences (Hosmer-Lemeshow Test).

**Table 18:** Logistic regression results for WTPEF in the HNP case.

Variables		β	S.E.	Wald	Exp. (β)	C.I. 95% Exp (β) Inf. Sup.	
P1	FEE (b)	−0.874 *	0.106	67.869	0.417	0.339	0.514
	Constant (a)	3.365 *	0.437	59.215	28.946		
Cox & Snell R^2^: 0.183; Nagelkerke R^2^: 0.350 (−2LL: 319.765) ** Overall correct percentage: 74.7%							
P2	FEE (b)	−0.830 *	0.150	30.576	2.292	1.708	3.076
	Constant (a)	1.879 *	0.314	35.798	0.153		
Cox & Snell R^2^: 0.165; Nagelkerke R^2^: 0.247 (−2LL: 260.674) ** Overall correct percentage: 69.7%							

Note: * Significant differences for a maximum error level of 1%; d.f. = 1 ** There are no significant differences (Hosmer-Lemeshow Test).

## Abbreviations

The following abbreviations are used in this manuscript:

NP	Natural Park
HNP	Hondo Natural Park
MTNP	Mata and Torrevieja Lagoons Natural Park
SPNP	Salt marshes of Santa Pola Natural Park
TCM	Travel Cost Method
CVM	Contingent Valuation Method
P_1_	Period 1
P_2_	Period 2
CS	Consumer Surplus
S1	Cost Scenario 1
S2	Cost Scenario 2
S3	Cost Scenario 3
FC	Fuel Cost
OTC1	Opportunity Travel Time Cost (0.06 €/km)
OTC2	Opportunity Travel Time Cost (0.14 €/km)
WAD	Weighted Average Distance
WAT	Weighted Average Time
WTP	Willingness to Pay
WTPEF	Willingness to Pay an Entrance Fee (logit analysis)
